# Fertilization of *Xenopus oocytes* using the Host Transfer Method

**DOI:** 10.3791/1864

**Published:** 2010-11-02

**Authors:** Patricia N. Schneider, Alissa M. Hulstrand, Douglas W. Houston

**Affiliations:** Department of Biology, University of Iowa

## Abstract

Studying the contribution of maternally inherited molecules to vertebrate early development is often hampered by the time and expense necessary to generate maternal-effect mutant animals. Additionally, many of the techniques to overexpress or inhibit gene function in organisms such as *Xenopus* and zebrafish fail to sufficiently target critical maternal signaling pathways, such as Wnt signaling. In *Xenopus*, manipulating gene function in cultured oocytes and subsequently fertilizing them can ameliorate these problems to some extent. Oocytes are manually defolliculated from donor ovary tissue, injected or treated in culture as desired, and then stimulated with progesterone to induce maturation. Next, the oocytes are introduced into the body cavity of an ovulating host female frog, whereupon they will be translocated through the host's oviduct and acquire modifications and jelly coats necessary for fertilization. The resulting embryos can then be raised to the desired stage and analyzed for the effects of any experimental perturbations. This host-transfer method has been highly effective in uncovering basic mechanisms of early development and allows a wide range of experimental possibilities not available in any other vertebrate model organism.

**Figure:**
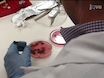


## Protocol

### 1. Surgical Removal of Ovary Tissue

1. Prepare a fresh batch of oocyte culture medium (OCM; Materials), between 400-800  mL depending on the size of the experiment. Adjust the pH to 7.6-7.8, until the phenol red turns a dark red color (six or so small drops of 5N NaOH). Pour out a small amount to check the pH in a separate tube since the pH electrode may contaminate the media. OCM should be stored at 18°C and used within a week.
2. Select 3-5 females and place one in ~ 2L buffered anesthetic solution (Materials). We try to avoid using frogs that were fed on the day of or prior to surgery. While the frog is succumbing to the anesthetic, disinfect the surgical area with 70% EtOH and prepare the surgical instruments.
3. Sterilize the surgical instruments by 20-second immersion in a hot bead sterilizer at 250°C. The following instruments should be on hand: scalpel handle (#3) and blade (#10 or 11), several pairs of Dumont forceps, Bonn iris scissors (curved or straight), Halsey or Olsen-Hegar micro needle holder, and sutures. Place sterilized instruments on a sterile Petri dish or on the inside of the suture package to keep sterile. Grasp the needle in the needle holder in the desired orientation before beginning. We prefer Olsen-Hegar needle holders since the handle contains scissor blades, which can be used to trim sutures without switching instruments.
4. After 10 minutes, check that a surgical plane of anesthesia has been achieved. Anesthetized frogs will stop moving and will not respond to a toe pinch or being turned over. Anesthesia should last 15 minutes, which is more than enough time to perform the operations. Don appropriate surgical attire. This can include clean gloves (non-latex), lab coats, and surgical masks, depending on personal preference and institutional animal care guidelines.
5. Note on aseptic technique: Frog skin secretions contain antimicrobial peptides that generally reduce the incidence of contamination during surgery, although aseptic technique should be followed as much as possible. Surgical drapes can be used, but may not be required by institutional IACUCs and may not provide much benefit. Generally "tip technique" used, where the hands do touch the sterile tips of the instruments, suture material or incision site. Instruments can be resterilized (in the bead sterilizer) following incision of the skin, and a sterile area should be maintained on the benchtop for placing instruments (such as a Petri dish or suture package). Institutional requirements can differ significantly, so please follow your institutions' guidelines and receive appropriate instruction prior to performing surgeries.
6. Remove the frog from anesthetic and place in dorsal recumbency on a damp surgical wipe. Note. The skin can be cleaned by rinsing in diluted povidine iodine (1:20) or chlorhexidine (0.75%). However, avoid commercial surgical scrubs, soaps and 70% isopropyl alcohol, as these will damage the delicate skin of amphibians.
7. Using a scalpel or small iris scissors, make a small (1- 1.5 cm) incision in the skin in the lower part of the abdomen, lateral to the midline. Subsequent incisions on the same frog should be performed on alternate sides, well spaced from each other. Incisions can be made either parallel or perpendicular to the midline.
8. Make the skin and abdominal muscle incisions in two stages. After cutting the skin, lift the muscle layer and surrounding white-colored fascia with forceps and make the incision in the raised muscle. Use scissors and cut straight down, try to make one swift cut to expose the body cavity leaving clean wound edges. If just the fascia is cut, it will retract and will make suturing more difficult. Lifting the muscle will help avoid inadvertently wounding any internal organs. Also, try to avoid cutting through any visible lymph hearts or blood vessels within the muscle layers. Extend the length with scissors of the incision so that ovary can be easily pulled through.
9. Ovary should be clearly visible once the incision is made. To procure the ovary, grasp with forceps and externalize the ovary tissue. The desired amount of tissue is removed, and trimmed off at the level of the body wall. Do not stuff the tissue back into the coelomic cavity, as this could be a source of infection or other complications.
10. Immediately observe a small piece of ovary under a dissecting microscope and defolliculate a few oocytes to test their quality. They should be firm and of uniform size and coloration. If they are acceptable, remove the desired amount of ovary tissue and suture the incision. Enough to fill a 90 mm Petri dish is usually sufficient for most experiments. If the oocytes are of questionable quality, suture the frog and repeat with a different female.
11. Close the incision by suturing the muscle and fascia layer first. Insert the needle a few millimeters lateral to the incision, making sure to pass through the fascia layer as well. Sutures just through the muscle may tear the tissue and come loose. Use forceps to help insert the needle from below on the opposite side of the incision. Release and re-grasp the needle from outside the body and pull the suture through until several inches or so of the tail remains, taking care not to pull it all the way through.
12. Close the wound with a simple interrupted pattern of suturing. We use a basic instrument tie to make surgeon's square knots (reef knots). Loop the long end of the suture around the needle holder, grasp the tail and pull it through the loop towards you and tighten. Loop and pull through again, this time pull the needle holder away from you. A third throw can be used for added security (in the same direction as the first).  Trim the suture, leaving short tail and do another suture in the muscle a few millimeters away. Two sutures are sufficient for small sutures; three for larger ones. Repeat for the skin layer.  Video demonstrations of basic suturing technique are available on YouTube -- search for "instrument tie"; there are quite a few to choose from, although the basic technique is the same.
13. Rinse off the female with deionized water and place her in a recovery bucket filled with cool (18°C) water. While in the lab, frogs are kept in tap water treated with Amquel to remove chloramines. Every few minutes, gently lift the frog from the water and look for gulping or eye-bulging movements, indicative of recovery from anesthesia.
14. Note: Survival surgery is not strictly necessary to obtain ovary tissue, although the females will continue to produce good oocytes after a number of operations. Performing survival surgery will also cut down on the numbers of animals used. Be sure to follow your animal care unit's guidelines on vertebrate survival surgery methods, post-op care and monitoring and for number of allowed surgeries on an individual.

### 2. Culturing and Defolliculating  Oocytes

1. Right after the surgery is finished, subdivide the ovary into small pieces (~2 cm^2^) and store in fresh OCM, using 5-6 pieces per 90 mm dish. Individual lobes of ovary are cut open, flattened out and cut into square pieces. These are rinsed in clean OCM and placed in dishes for culture. Flattening and dividing up the ovary will extend its life in culture and make defolliculation easier.
2. Move a lobe to a small dish of OCM and manually defolliculate 300-500 oocytes. Transfer oocytes using a sterile, fire-polished pipette. We have found that using the cotton-plugged variety is essential for minimizing contamination of the cultures. Store the oocytes at 18°C in 8  mL OCM (in medium dishes (Falcon 1007)) in groups of 100-150. With practice, you should be able to defolliculate enough oocytes in 1-2 hours. Collagenased oocytes are not suitable for host transfer since they are not be fertilizable.
3. Defolliculating method. We defolliculate using watchmakers' forceps (Dumont #4s or #5s, Fine Science Tools), following the basic methods described in Smith *et al.*, (1991). Using one pair of forceps (in your dominant hand), grasp the theca connective tissue layer surrounding a fully-grown stage VI oocyte near where the follicle is attached to the ovary (the stalk). With the other pair of forceps, grasp the ovary adjacent to the first pair. Lightly tear open the follicle layer by pulling across the oocyte.  Gently tease the oocyte out of the tissue. Successfully defolliculated oocytes are floppier than oocytes simply pulled off without removing the follicle layer and will be devoid of visible blood vessels.
4. Note: It is essential to maintain very light pressure on the tips of the forceps, barely enough for the tips to meet. Gripping too tightly is a common among beginners and will result in the oocyte being pulled away with the follicle intact. Keep a good pair of forceps exclusively for defolliculating. The tips should meet precisely and should be slightly blunter than forceps for making explants from *Xenopus* embryos (Figure 1).
5. We routinely test mature about 20 with progesterone and abort the experiment if the maturation rate is poor (<50% after 6-7 hours at room temperature).
6. At this point, the oocytes can be manipulated and cultured as desired, such as microinjection of oligo, morpholino or mRNA. Injection procedures vary per lab, so we will not describe those here. Oocytes can be maintained up to a week in OCM, but generally the health declines so it is best to use them for transfer within 96 hours of isolation.
7. Keep in mind that only five-to-six groups (including a control) can be transferred to a single female, owing to limitations on vital dyes, so plan your experiments accordingly.

### 3. Oocyte Maturation and Stimulation of Prospective Host Females

1. If performing a rescue of oligo depletion, inject your mRNA (usually 50-300 pg of RNA) ~24-48 hours after the oligo. The oligos will have degraded by this time and should not affect the injected RNA.
2. On the evening prior to performing the transfer, add 16  μL of progesterone working solution (1mM in EtOH) to oocytes in 8  mL OCM in medium dishes (final progesterone concentration is ~2 μM). Incubate 10-12 hours at 18°C to allow for oocyte maturation.
3. Inject 3-5 females with 1000 units per frog of hCG to induce egg laying. Our experiments seem to work better if oocyte maturation and hCG injection are done around the same time, about 12 hours prior to implantation into the host (e.g., 10 PM -10 AM). Leave females at RT or 18°C overnight.

### 4. Preparation and Vital Dye Staining of Oocytes

1. On the morning of the transfer, allow ~ 45-60' to set up and perform the procedure. At this point, mature oocytes can be frozen for later analysis of the efficacy of mRNA knockdown or protein expression. Also, check that the oocytes have not become infected and that there was a good maturation rate (>50%). Infected oocytes will not fertilize. Thaw vital dyes (Materials) and spin at max speed for 5 min.
2. Color the different experimental groups by adding 80  μL of the appropriate vital dye(s) to the oocyte dishes and incubate with rocking for 15'. During this time, select a host female and begin anesthesia. Choose a frog that is slowly laying eggs normally or one that produces abundant eggs with mild squeezing. Eggs from the host should be of good quality. Avoid females that are laying strings of eggs or crushed eggs.
3. Transfer the colored eggs to a large dish of fresh OCM, swirl briefly to wash and set them aside prior to transfer.
4. Prepare a sterile Pasteur pipette with a bore just wide enough to accommodate an oocyte (~ 1.5 mm) for use in transplanting the oocytes. Score the pipette with a diamond pencil and break cleanly. Fire polish until the edges are smooth, taking care not to melt the opening closed.

### 5. Performing the Oocyte Transplantation

1. Oocyte transplantation uses the same surgical procedures as described in Part I, although instead of removing ovary, manipulated oocytes are introduced into the body cavity with a pipette. Ideally, the transfer procedure should be performed as rapidly as possible and the size of the incision should be kept as small as possible.
2. Anesthesize the host female and prepare surgical instruments as described in Part I. Make and incision as described in Part I. The incision can be smaller, but needs to be large enough to fit the bore of the transfer pipette (Figure 2A).
3. To transplant the oocytes, grasp and elevate one flap of muscle and fascia with forceps and introduce the experimental oocytes using the Pasteur pipette prepared above. Swirl the dish of oocytes to collect them into the center and suck up as many as possible. Allow the oocytes to settle into the end of the pipette to prevent excess liquid from being introduced and insert the tip of the pipette into the incision and slowly allow the oocytes to trickle into the body cavity (Figure 2B). Repeat until all oocytes have been transferred. Do not release the muscle layer at any time, until you have begun suturing, or the oocytes will spill out of the incision.
4. Once the oocytes have been transferred, grasp both sides of the incision with forceps and bring them together, allowing the oocytes to settle into the body cavity. Begin suturing as described, taking care to keep upward tension on the muscle and fascia until the first knot is tied (Figure 2C). This will keep the oocytes from being expelled out and will allow the edges of the incision to heal evenly. Add another suture in the muscle/fascia layer and then suture the skin.
5. Rinse off the female with deionized water and place her in a recovery bucket filled with cooled (18°C) Amquel-treated water. Monitor her recovery from anesthesia as above. She should begin laying eggs normally after the operation.
6. Obtain testes from a male frog using normal procedures. Store the testes in OCM or L15 until needed. We prefer fresh testis when fertilizing transfer experiments.

### 6. Recovery of Oocytes and *in vitro* Fertilization

1. Peritoneal cilia will drive the transferred eggs (and normal coelomic eggs of the host) to the openings of the oviducts in the anterior of the body cavity. Colored eggs can be recovered beginning 2-3 hours after the transfer by normal squeezing. Eggs can be obtained every half-hour or so until the female stops laying.
2. Fertilize in a sperm suspension (in ~ 4 mL 1x MMR) for 4 minutes. Flood and rinse extensively with 0.1x MMR. Eggs should activate normally and will begin to cleave ~ 1.5-2 hrs after fertilization. Transferred eggs will often cleave slightly later than the host eggs.
3. Remove the jelly coats with cysteine as normal and sort embryos into separate dishes. This can be done at any stage, depending on the needs of the experiment. It usually easiest to identify the various colors around the 4-cell stage and most difficult during late blastula stages. Culture the eggs at a low density (< 50 per medium dish) in clean 0.1x MMR. From this point, embryos can be treated and manipulated normally.
4. Return the frogs to the animal facility and monitor for complications over the next few days.
5. Alternate fertilization method; egg-laying in high salt buffer
  1. After the frog has recovered from anesthesia, place her into ~ 1L high-salt MMR (1.2x). Allow the female to lay eggs into the buffer for about 4-6 hours. Squeeze remaining eggs out, drain 1.2x MMR and sort colored eggs into clean dish; remove all possible liquid.
  2. Wash extensively with 0.3x MMR and fertilize in minimal volume of 0.3x MMR/sperm suspension until eggs activate (10 minutes). Flood with 0.1x MMR and culture as above. This option is useful if many eggs are needed at the same stage or if manual expression of eggs damages the donor oocytes.

## Discussion

A successful transfer will result in fertilization and normal cleavage of > 50% of the oocytes (Figure 3). Generally between 30-60% of the transferred oocytes will survive past the neurula stages. We usually transfer 75-150 oocytes per experimental group, which will yield enough embryos for several types of analysis (in situ, RT-PCR) as well as visualizing the phenotype. Implanting substantially more oocytes does not seem to greatly increase the yield. Also, at least 30 oocytes per group should be transferred to guarantee that at least some make it through gastrulation. The vital dyes generally do not affect the embryos under the concentrations and conditions described here, although dyes may have adverse effects in certain situations. Classically, Neutral Red has been reported to have phototoxic properties when exposed to strong tungsten based light sources. More recently, Mir and Heasman (2008) have reported that Bismarck Brown can have some toxic effects if the embryos are incubated at low temperatures.  Basic precautions such as not excessively dying the oocytes and avoiding extremes of temperature and illumination should eliminate the possibility of vital dye side effects.

The greatest determinants in the success or failure of the method are the quality of the donor oocytes and the host female. There is no foolproof method for determining whether a particular batch of oocytes will fertilize well. Ovary with a high proportion of atretic oocytes should be avoided.  Also, we have had poor success with ovary that is heavily vascularized, possibly indicating that resorption is occurring. Oocytes should be kept at 18°C as much as possible and otherwise kept in healthy condition as oocytes will not fertilize if infected or damaged. It also appears, in our lab, that better results are achieved when the defolliculator is more experienced and can isolate and inject large numbers of oocytes in a short time frame. Similarly, care should be taken to select hosts with healthy eggs, although it is never certain that a given frog will make a good host. As with defolliculating, the skill of the surgeon also has a bearing on the success of the procedure. Minimizing the length of time the host is anesthesized seems critical to efficient recovery and fertilization.

The overall timing of the various procedures is critical to successful oocyte transfer experiments. In general, the shorter the time oocytes are kept in culture, the better the overall fertilization and health of the embryos will be. For mRNA knockdown experiments using antisense oligos, oocytes should be cultured at least 24 hours after oligo injection to allow for maximal depletion of the target mRNA and turnover of the oligo. Thus oocytes should be defolliculated as soon as possible and injected the same day. A typical experiment might begin on Monday with oocyte isolation and injection, followed by maturation and stimulation of females on Tuesday evening, and transfer on Wednesday morning. If the target RNA turnover is slow or there is abundant maternal protein, the culture period can be extended to 48 or even 72 hours. We have the best success when the oocytes are transferred within 12 hours of treating with progesterone. Over-mature oocytes begin to deteriorate and generally fertilize poorly. We generally add progesterone and inject females with hCG about 12 hours before performing the transfer, with both oocytes and frogs kept at ~ 18°C overnight.

Although the host-transfer method is labor intensive initially, the basic necessary skills can be acquired without much difficulty. The most common use for this method is to fertilize oocytes that were depleted of specific maternal mRNAs by antisense oligonucleotide injection. Other manipulations such as mRNA overexpression or drug treatment can also be performed. These can often have different results compared with injection following fertilization, owing to increased expression or differential function in eggs versus embryos. E-cadherin overexpression (Heasman *et al.*, 1991) and  ultraviolet irradiation (Holwill *et al.*, 1987; Elinson and Pasceri, 1989) are two interesting examples. Preliminary results from our lab also suggest that transgenesis methods using injected integrase or meganucleases may work more efficiently in oocytes as well.

The host transfer technique allows experimentation at both pre- and post-fertilization stages and thus can provide insights not possible in other organisms or even by looking solely at post-fertilization events in *Xenopus*. Given the importance of maternal signaling pathways in development and difficulty and cost of generating maternal effect mutations in vertebrates, this method is likely to remain a useful tool in the investigation of early development.

### 7. Representative Results 


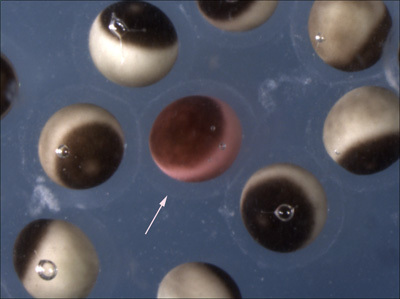
 **Figure 1. Examples of good (top) and bad (bottom) forceps for defolliculating.**


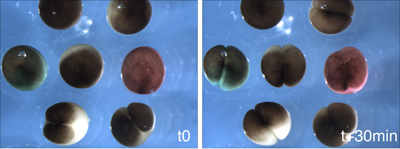
 **Figure 2. Transfer of cultured oocytes into a host female.** (A) a small incision is made in the lower abdomen of the host frog. The muscle layer is elevated slightly to allow introduction of the oocytes. (B) oocytes are vital dyed and placed in the body cavity through the incision. (C) completion of the initial suture in the muscle layer.


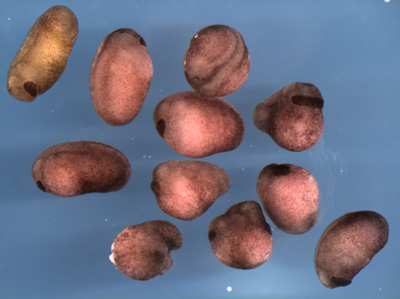
 **Figure 3. Examples of dividing, transferred oocytes at the 8-16-cell stage, following removal of the jelly coats.**

## References

[B0] Brun R (1975). Oocyte maturation in vitro: contribution of the oviduct to total maturation in Xenopus laevis. Experientia.

[B1] Elinson RP, Pasceri P (1989). Two UV-sensitive targets in dorsoanterior specification of frog embryos. Development.

[B2] Heasman J, Holwill S, Wylie CC, C C (1991). Fertilization of cultured Xenopus oocytes and use in studies of maternally inherited molecules. Methods Cell Biol.

[B3] Holwill S, Heasman J, Crawley C, Wylie CC (1987). Axis and germ line deficiencies caused by u.v irradiation of Xenopus oocytes cultured in vitro. Development.

[B4] Houston DW, King ML (2000). A critical role for Xdazl, a germ plasm-localized RNA, in the differentiation of primordial germ cells in Xenopus. Development.

[B5] Mir A, Heasman J (2008). How the mother can help: studying maternal Wnt signaling by anti-sense-mediated depletion of maternal mRNAs and the host transfer technique. Methods Mol Biol.

[B6] Rugh R (1962). Experimental Embryology: Techniques and procedures.

[B7] Smith LD, Ecker RE, Subtelny S (1968). In vitro induction of physiological maturation in Rana pipiens oocytes removed from their ovarian follicles. Dev Biol.

[B8] Smith LD, Xu W, Varnold RL (1991). Oogenesis and oocyte isolation. Methods Cell Biol.

[B9] Woolf TM, Jennings CG, Rebagliati M, Melton DA (1990). The stability, toxicity and effectiveness of unmodified and phosphorothioate antisense oligodeoxynucleotides in Xenopus oocytes and embryos. Nucleic Acids Res.

[B10] Wylie C, Kofron M, Payne C, Anderson R, Hosobuchi M, Joseph E, Heasman J (1996). Maternal beta-catenin establishes a 'dorsal signal' in early Xenopus embryos. Development.

[B11] Zhang J, Houston DW, King ML, Payne C, Wylie C, Heasman J (1998). The role of maternal VegT in establishing the primary germ layers in Xenopus embryos. Cell.

[B12] Zuck MV, Wylie CC, Heasman J, Richter JD (1998). Maternal mRNAs in Xenopus embryos: an antisense approach.. A comparative methods approach to the study of oocytes and embryos.

